# Endoscope-assisted low-temperature plasma radiofrequency ablation of intermuscular angiolipoma in the chest: a case report

**DOI:** 10.3389/fped.2025.1660856

**Published:** 2025-10-31

**Authors:** Yaoyao Li, Ming Zhao, Xin Wang, Jian Rong, Jianing Wang, Mei Song

**Affiliations:** ^1^ Department of Burns and Plastic Surgery, 940th Hospital of Joint Logistics Support Force, People's Liberation Army, Lanzhou, China; ^2^ Department of Plateau Medicine, 940th Hospital of Joint Logistics Support Force, People's Liberation Army, Lanzhou, China; ^3^ First Clinical Medical College, Gansu University of Chinese Medicine, Lanzhou, China

**Keywords:** endoscope-assisted surgery, low-temperature plasma radiofrequency ablation, intermuscular angiolipoma, angiolipoma, surgery

## Abstract

**Background:**

Endoscope-assisted surgery (EAS) remains largely unexplored for intermuscular angiolipomas (IA) in children. This study reports a 6-year-old girl with a right chest angiolipoma who underwent EAS via a minintermuscular angiolipomaly invasive anterior axillary fold approach.

**Methods:**

Following ultrasonographic and magnetic resonance imaging revealing a provisional diagnosis of IA, the patient underwent endoscope-assisted low-temperature plasma radiofrequency ablation (LTPRA) under general anesthesia. The procedure was performed through a single 2 cm incision at the right anterior axillary fold utilizing a 5 mm 30° endoscope and pediatric-specific fine instruments.

**Results:**

Complete resection of the angiolipoma was achieved with an operative time of 120 min and blood loss of 15 ml. Pathological examination confirmed angiolipoma. At 6-month follow-up, no recurrence and satisfactory wound healing have been observed.

**Conclusion:**

EAS proves a feasible and effective therapeutic option for complex anatomical-site angiolipomas due to its efficacy in complete excision and superior cosmetic outcomes. By integrating endoscopic visualization with precise plasma ablation, this technique significantly reduces neurovascular injury risks during deep intermuscular tumor dissection, offering a novel minintermuscular angiolipomaly invasive strategy for lesions in regions challenging for conventional open surgery.

## 1 Introduction

Angiolipoma (AL) is a relatively rare benign mesenchymal tumor characterized by slow growth and typically asymptomatic presentation. However, when enlarging in size or compressing adjacent neurovascular structures, it may induce pain, motor dysfunction, or space-occupying effects (1–3). In the pediatric chest wall, fat-containing tumors with vascular components are uncommon and require careful preoperative counseling and planning to balance oncologic safety with preservation of function and cosmesis during growth (4, 5). Particularly for intermuscular angiolipoma (IA), their deep anatomical location and ill-defined boundaries with surrounding muscle tissue make traditional open surgery prone to extensive muscle injury, postoperative hematoma, and neurological complications.

Magnetic resonance imaging (MRI) is central to preoperative assessment because it can depict both macroscopic fat and vascular elements that characterize AL/IA. Helpful clues include fat-suppressed signal drop, internal thin septations, flow-voids, and dynamic contrast-enhancement behavior, which together help differentiate angiolipoma from simple lipoma, lipoblastoma, intermuscular lipoma, and liposarcoma mimics in children; they also inform the operative corridor and anticipated resection plane (6–8). These imaging cues supported our impression of a fat-rich vascular lesion within an intermuscular plane and guided the surgical approach.

The advancement of endoscope-assisted surgery (EAS) has paved the way for minimally invasive management of deep, narrow intermuscular spaces in the pediatric chest wall. When paired with low-temperature plasma radiofrequency (RF), EAS enables precise tissue ablation with limited thermal spread and concurrent hemostasis, advantages near developing musculature and neurovascular structures compared with conventional electrocautery or open exposure (9, 10). Contemporary pediatric series and technique reports across airway/head–neck and thoracic contexts show reduced bleeding, less postoperative pain, and favorable recovery with endoscopic RF, supporting our choice of an endoscope-assisted plasma strategy for this intermuscular lesion (9, 10).

We present a case of a 6-year-old girl presented who presented with a progressively enlarging subcutaneous mass in the right chest wall over five years. Imaging studies confirmed the diagnosis of IA. The patient underwent successful endoscopic-assisted tumor resection via a posterior intermuscular approach, combined with piecemeal ablation using plasma ablation technology.

## 2 Case presentation

**Figure 1:**
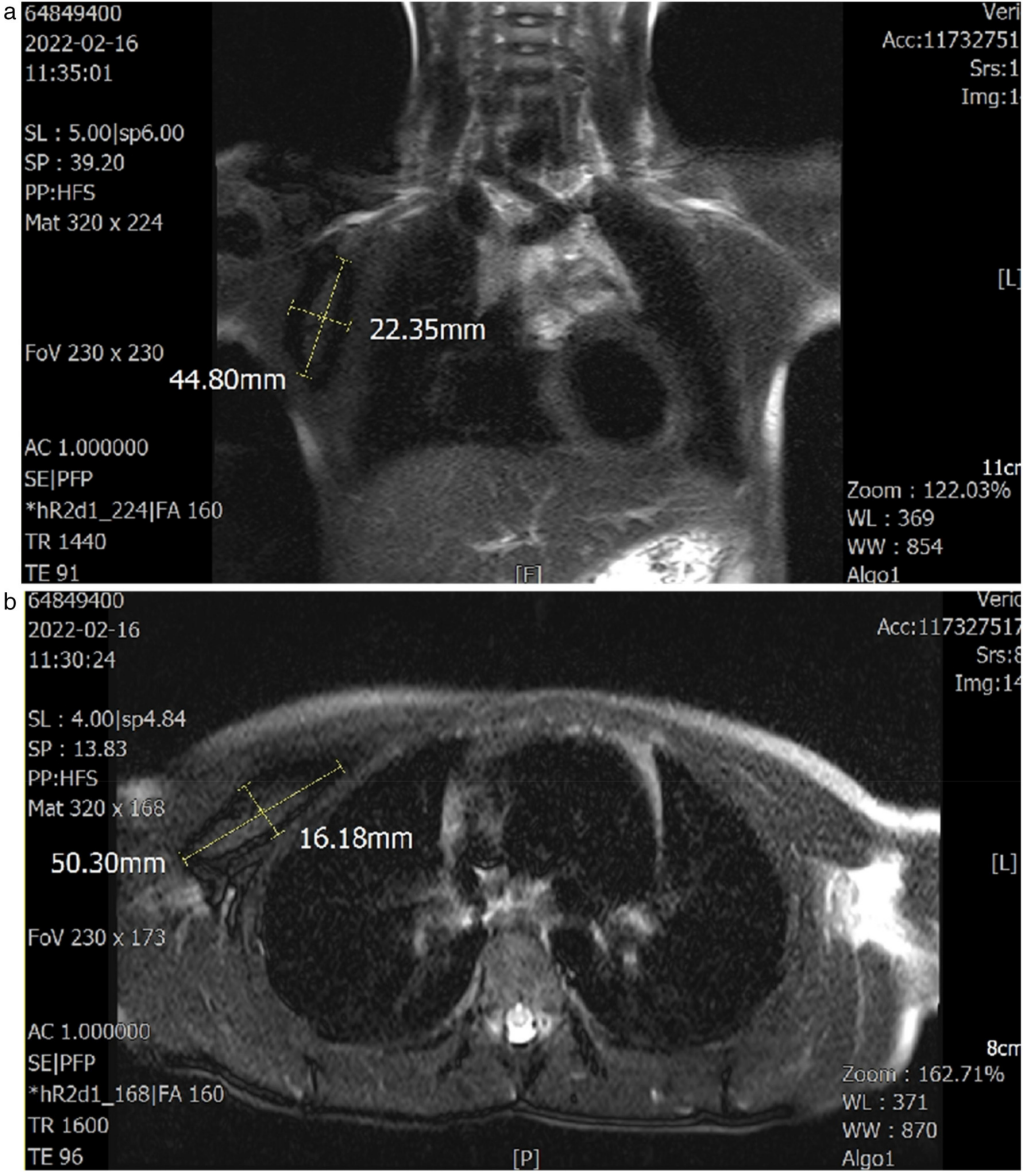
**(a,b)** Axial and coronal T1- and T2-weighted images demonstrate the lesion site and dimensions. The sagittal plane was unremarkable.

A 6-year-old girl patient was admitted to our hospital with a 5-year history of progressive enlargement of a right chest subcutaneous mass. General physical examination and blood biochemical tests were normal, with unremarkable personal history. Chest MRI demonstrated a lobulated slightly short T1 signal mass in the intermuscular space between the right pectoralis major and minor muscles. The lesion exhibited peripheral low signal on fat-suppressed T2-weighted imaging (fs-T2WI) with patchy central hyperintensity, measuring approximately 2.3 × 5.0 × 5.5 cm in maximum cross-section. Diffusion-weighted imaging (DWI) revealed isointense to slightly hyperintense signal characteristics (Figure 1). These imaging features supported the diagnosis of IA in the pectoral muscle space.

**Figure 2:**
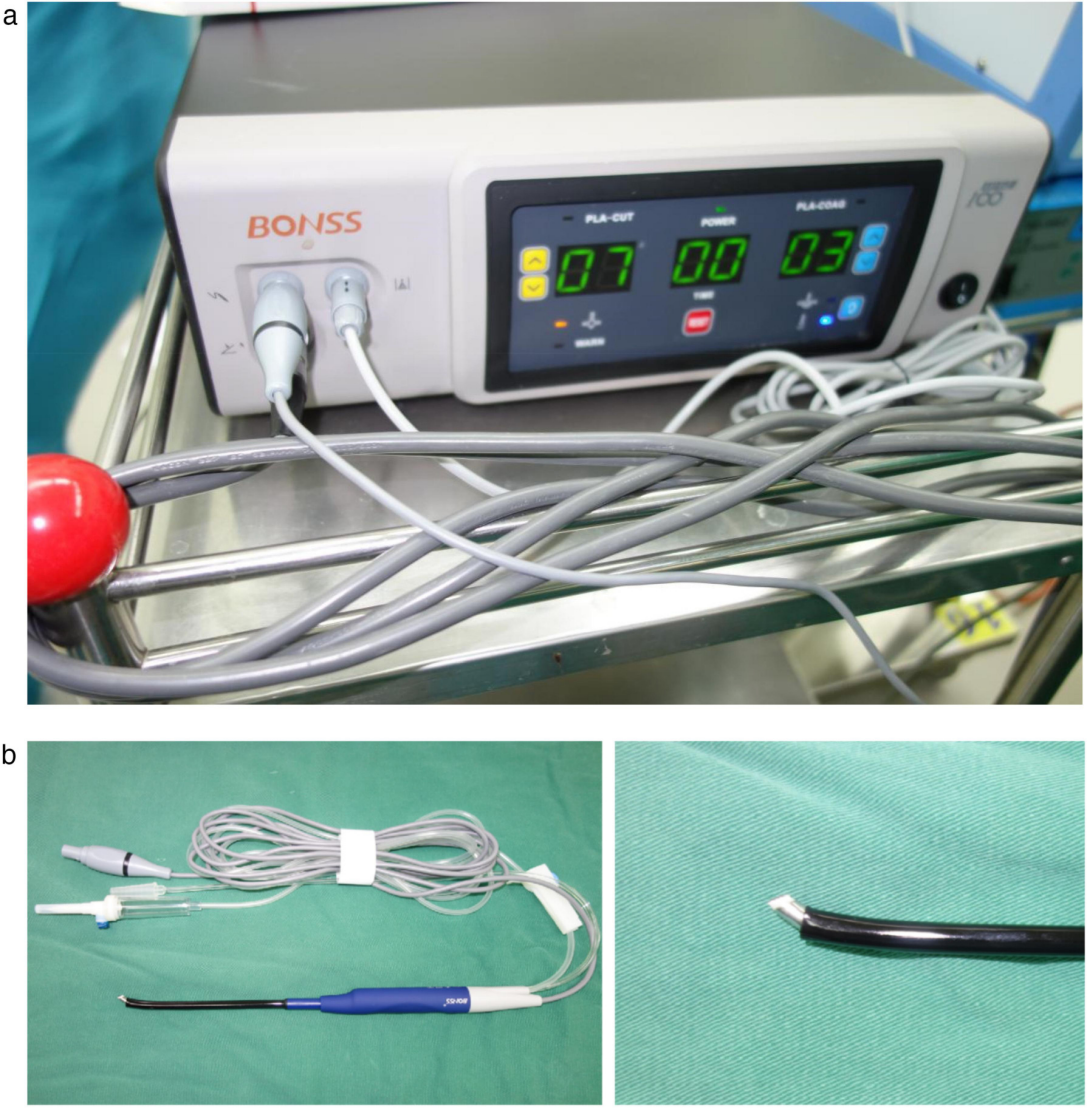
**(a)** Photograph of the LTPRA generator. **(b)** Low-temperature plasma knife and tip close-up.

Considering the patient's young age, large tumor size, and proximity to mammary gland and chest muscles, conventional large axillary incision might result in scarring and potential breast development impairment. While periareolar incision risked limited surgical exposure, glandular damage, and muscle dissection. After multidisciplinary team discussion, we elected to perform minintermuscular angiolipomaly invasive resection using endoscope-assisted LTPRA to achieve complete tumor removal while preserving functionality and optimizing cosmetic outcomes (Figure 2).

**Figure 3:**
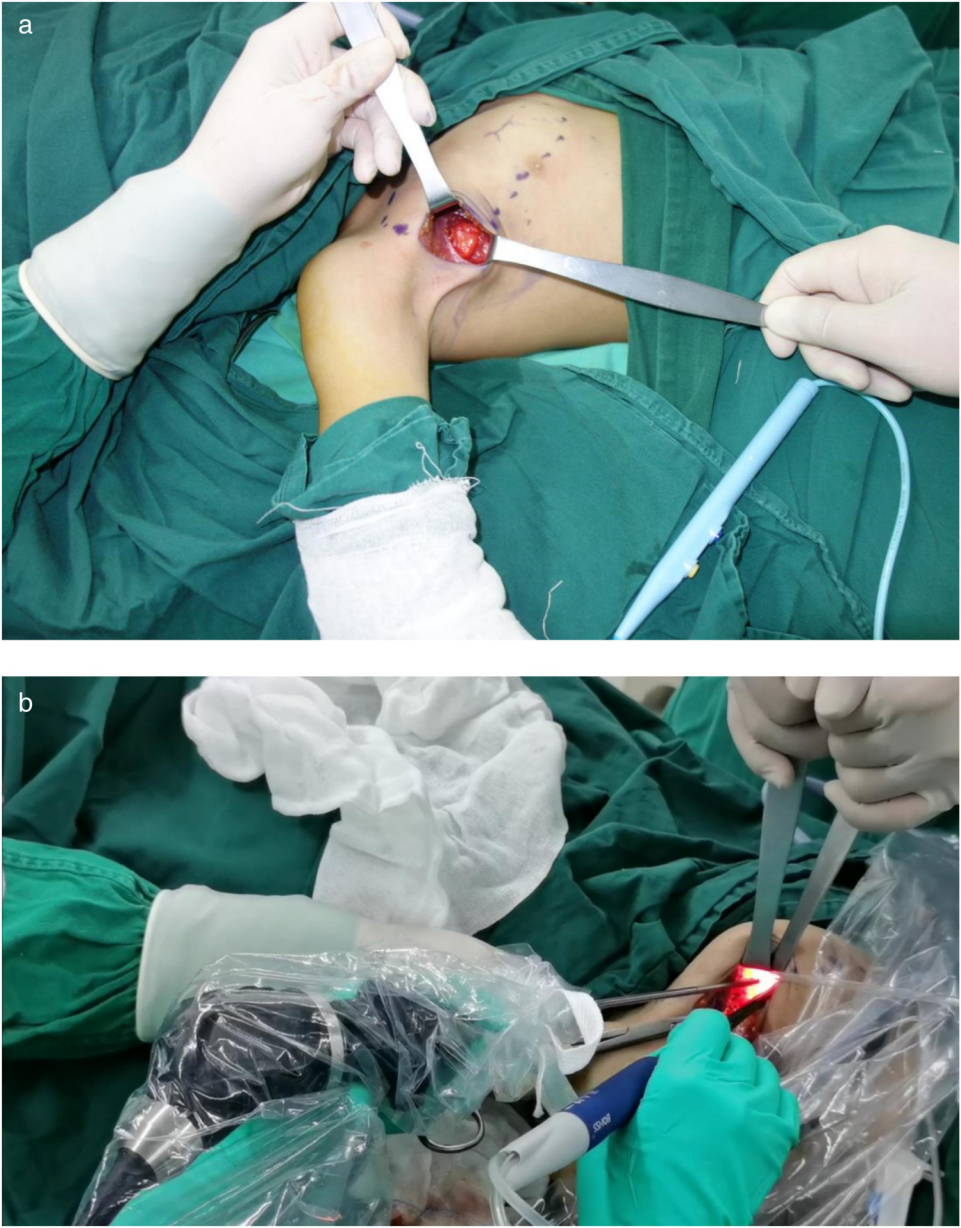
**(a)** Creation of the intermuscular working space. **(b)** A photo of the intraoperative trocar position.

**Figure 4:**
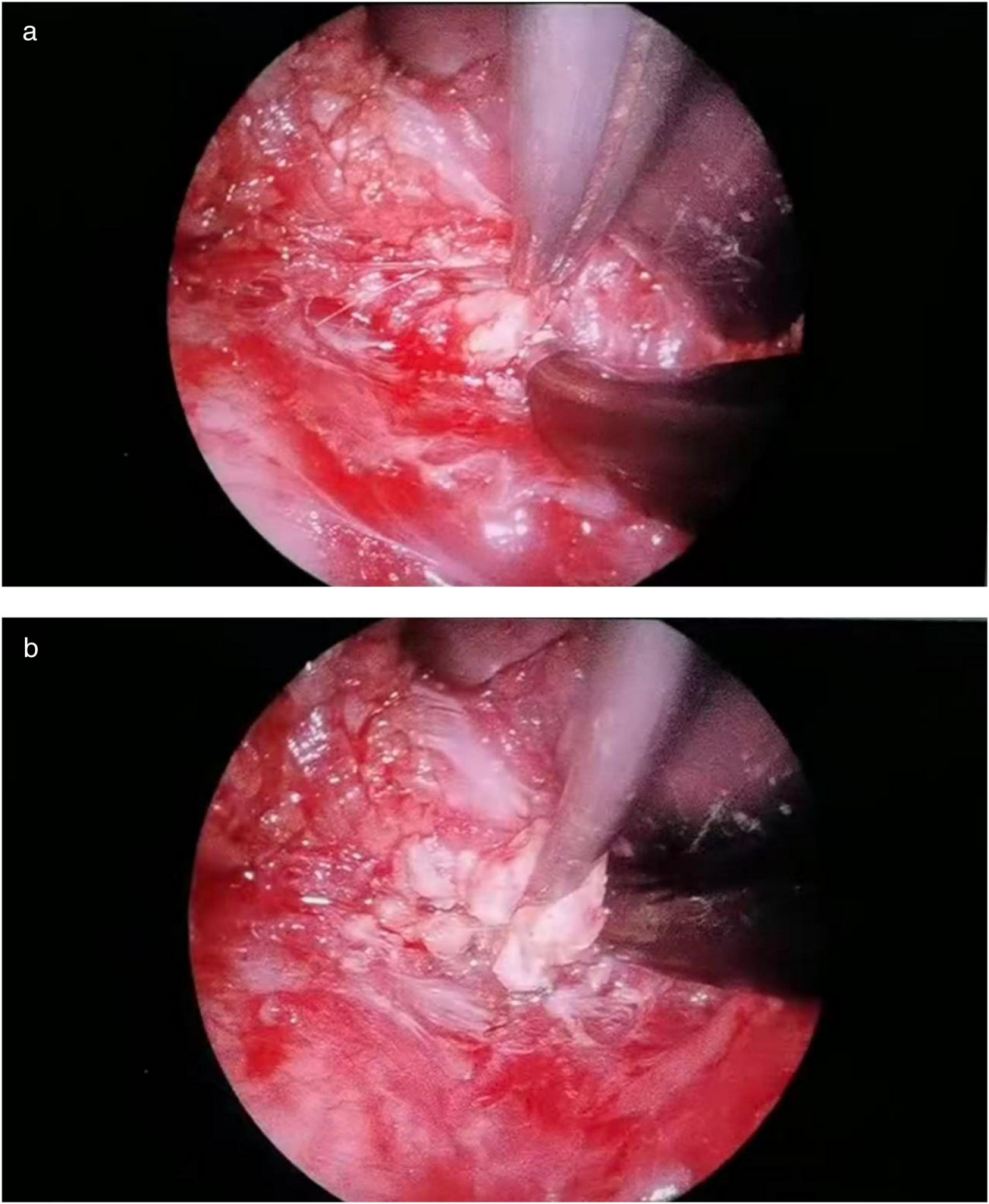
**(a,b)** Plasma ablation at the tumor–muscle interface with hemostasis.

**Figure 5:**
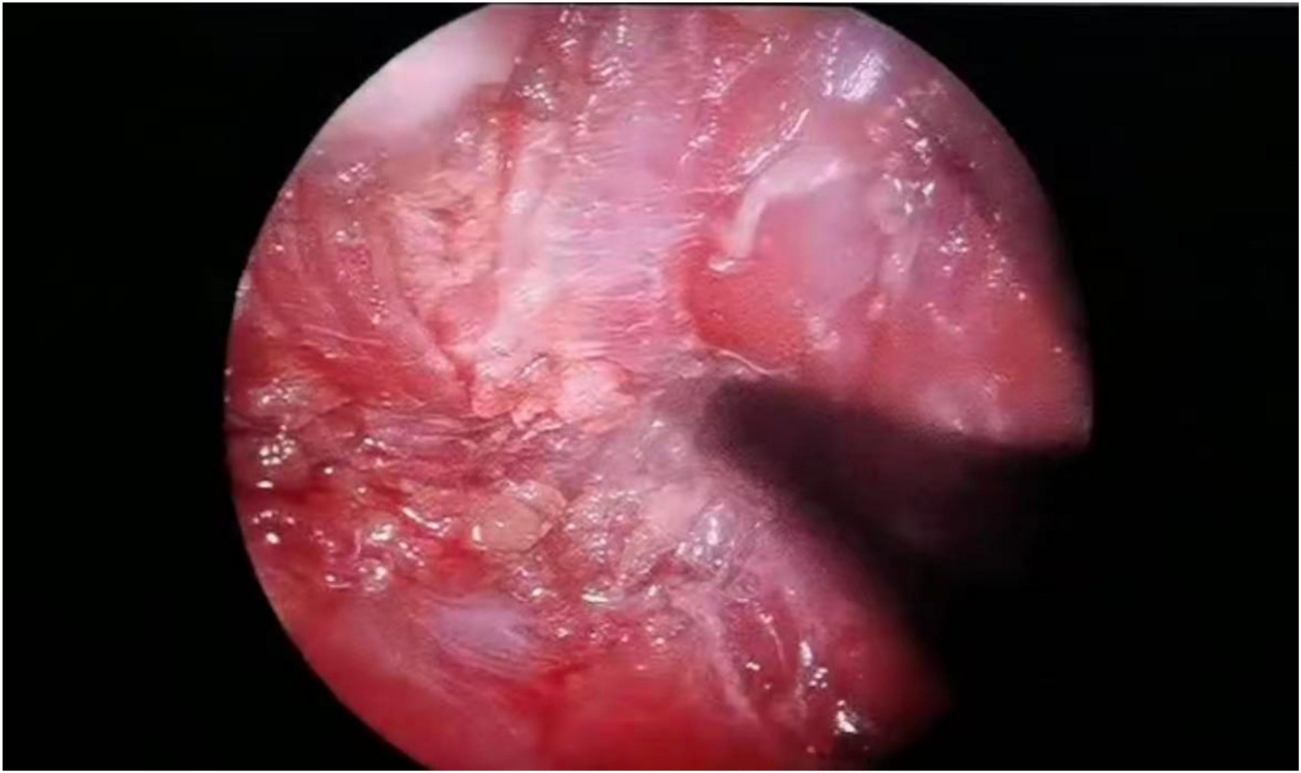
Perforation and nerve injury were ruled out.

**Figure 6:**
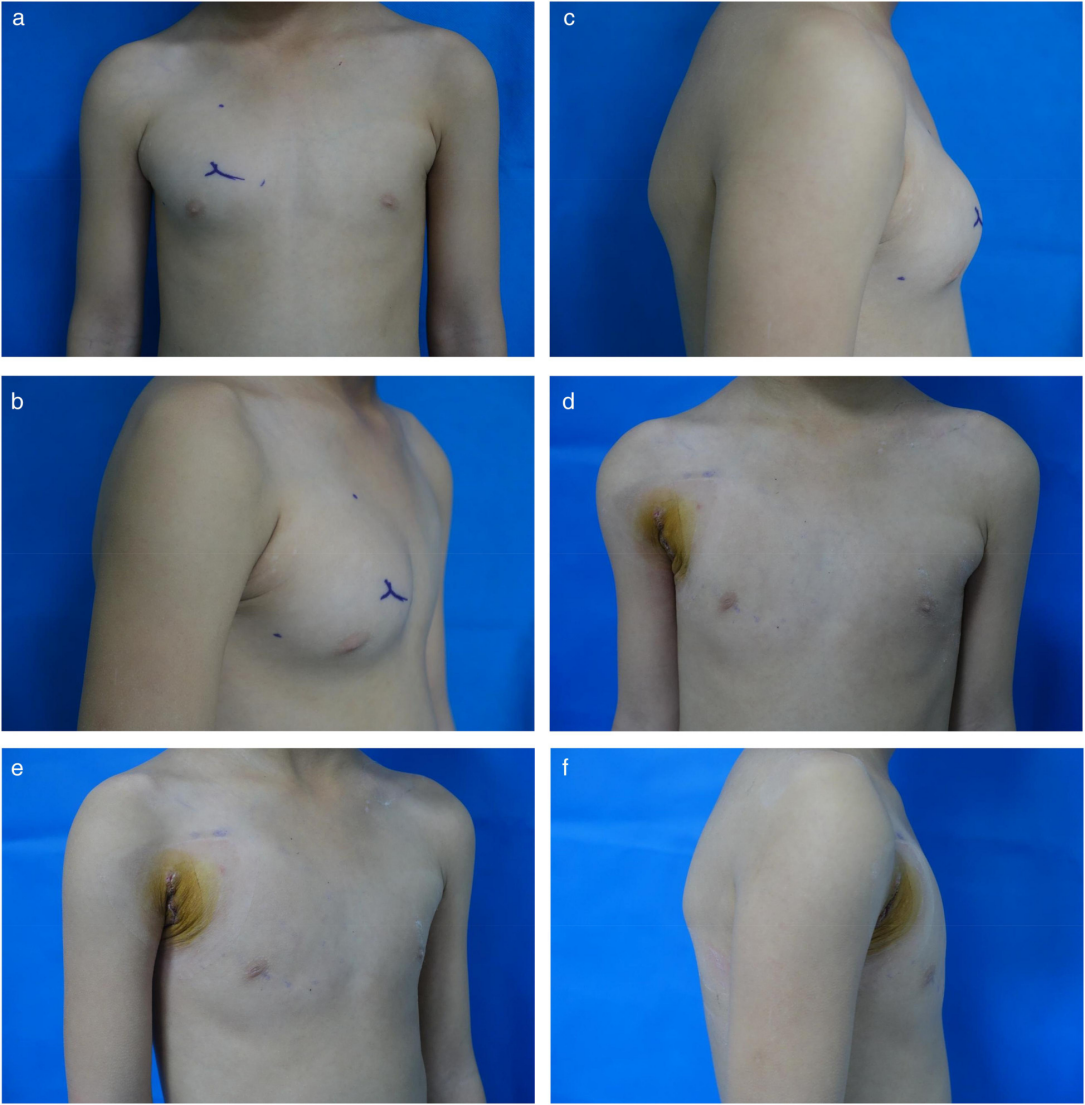
**(a)** Preoperative anteroposterior view. **(b)** Preoperative oblique view. **(c)** Preoperative lateral view. **(d)** 7-day postoperative anteroposterior view. **(e)** 7-day postoperative oblique view. **(f)** 7-day postoperative lateral view.

**Figure 7:**
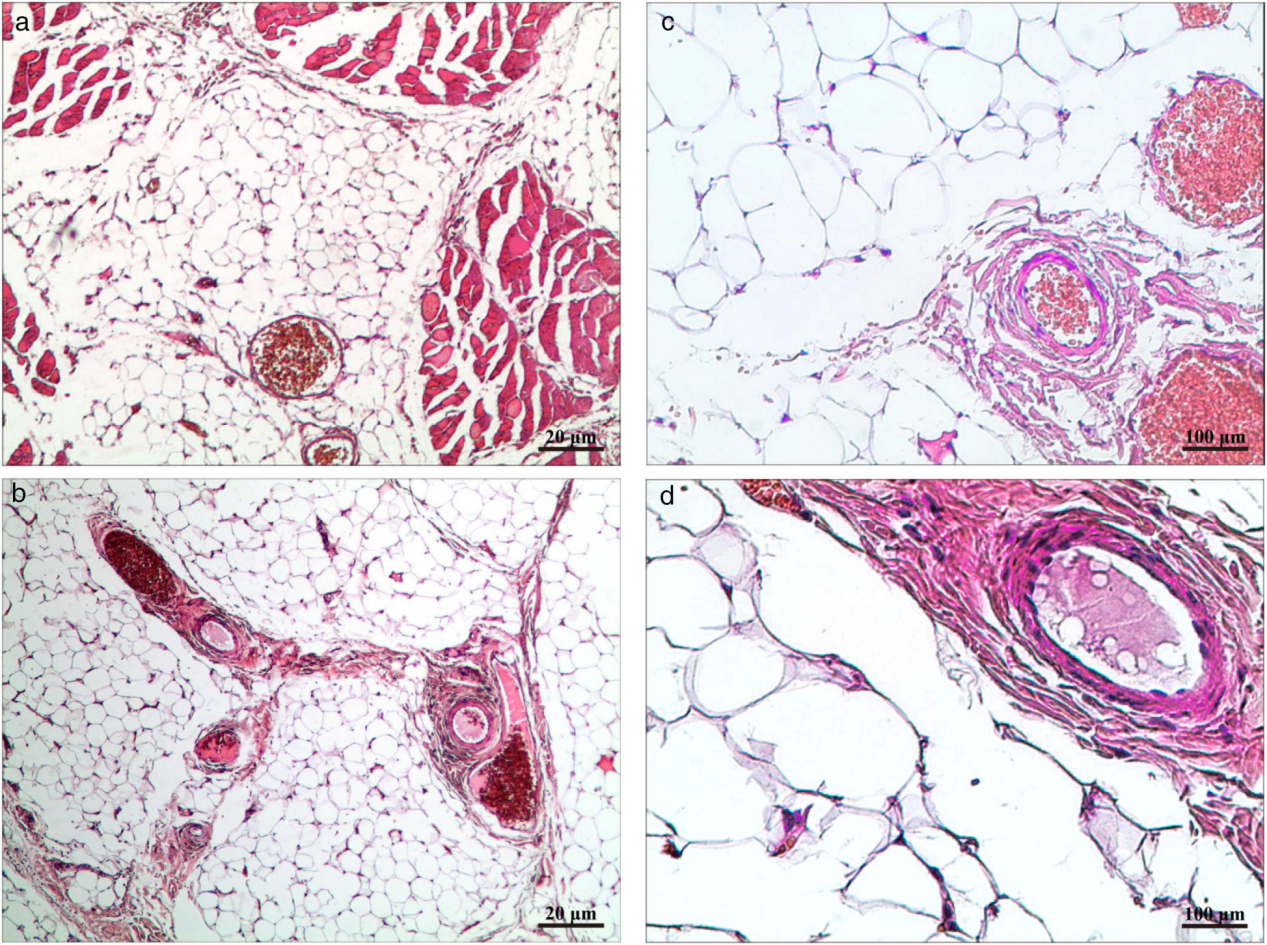
**(a,b)** HE × 40 highlighting mature adipocytes with interspersed small vessels. **(c,d)** HE × 200 showing proliferative small vessels with occasional fibrin thrombi and no cytologic atypia.

Through a 2 cm incision along the right anterior axillary fold, we sequentially incised skin and subcutaneous tissue to expose the lateral border of pectoralis major (Figure 3A). After dissecting the fascia to enter the retropectoral space, endoscopic separation of the intermuscular space between pectoralis major and minor was performed to establish an optical operative cavity (Figure 3B). The plasma radiofrequency knife was used to gradually dissect the tumor extending from the axillary inferior border to the medial nipple, medially adjacent to the sternum and laterally involving the lateral border of pectoralis major(Figure 4). The intact tumor specimen was sent for histopathological examination, with no residual lesion or active bleeding observed intraoperatively (Figure 5). The operation was successfully completed with an intraoperative blood loss of 15 ml and an operative time of 2 h. Within the postoperative period, the drainage tube discharged 8 ml and 3 ml of light red fluid, respectively. The drainage tube was removed 48 h after surgery, and the surgical site was managed with pressure dressing for 7 days. During wound care, the incision was observed to have healed well without infection, seroma, or necrosis. Sutures were removed on the 7th postoperative day (Figure 6). Postoperative histopathology confirmed the diagnosis of IA (Figure 7). At 6-month follow-ups, there was no tumor recurrence. The axillary incision scar appeared fine, white, and soft, with symmetrical bilateral thoracic contour and satisfactory functional and cosmetic outcomes.We summarized the care-episode timeline—including symptom onset, imaging, intervention, and outcomes—in Table 1.

**Table 1:** Care-episode timeline.

Event	Timing	Key data
Symptom onset	≈5 years before surgery	6-year-old girl; progressive enlargement of a right anterior chest subcutaneous mass; normal general physical exam and blood biochemistry; unremarkable personal history.
Pre-operative MRI (Figure 1)	Pre-op	Lobulated, slightly short-T1 mass in the intermuscular space between right pectoralis major and minor; fs-T2WI: peripheral low signal with patchy central hyperintensity; DWI: iso- to slightly hyperintense; size ≈2.3 × 5.0 × 5.5 cm. Imaging favored IA.
Surgery—endoscope-assisted LTPRA (Figures 3a, 4)	Day 0	2 cm incision along right anterior axillary fold; fascia opened to retropectoral space; endoscopic separation between pectoralis major/minor to establish optical cavity; plasma RF dissection from axillary inferior border to medial nipple; medially near sternum, laterally at lateral border of pectoralis major. Intact specimen sent for histopathology (Figure 5). EBL 15 ml; operative time 2 h. No residual lesion or active bleeding.
Post-op drainage	POD 0–2	Drainage 8 ml then 3 ml of light-red fluid; drain removed at 48 h; pressure dressing for 7 days.
Early wound status (Figure 6)	POD 7	Incision healed well without infection, seroma, or necrosis; sutures removed on POD 7.
Histopathology (Figure 7)	Post-op	Histopathology confirmed IA.
Follow-up outcome	6 months post-op	No tumor recurrence; axillary scar fine/white/soft; symmetric bilateral thoracic contour; satisfactory functional and cosmetic outcomes.

Surgery is designated as Day 0; other events are expressed as relative days when exact dates are unavailable. Abbreviations: IA, intermuscular angiolipoma; LTPRA, low-temperature plasma radiofrequency ablation; POD, postoperative day; EBL, estimated blood loss.

## 3 Discussion

AL is a rare benign tumor composed of mature adipose tissue and abnormally proliferative blood vessels, predominantly involving subcutaneous tissues of the trunk and extremities (1–3). In this case, the tumor exhibited infiltrative growth within the intermuscular space between the right pectoralis major and minor muscles, invading adjacent chest wall muscular structures with complex anatomical relationships. Although histopathological examination remains the diagnostic gold standard, precise preoperative evaluation is crucial for surgical planning.

EAS refers to the integration of endoscopic visualization for manipulating anatomical regions difficult to access through direct vision in conventional open procedures. For AL management, traditional open approaches typically require lengthy incisions parallel to skin tension lines over the lesion surface, which may lead to extensive muscle dissection, significant intraoperative blood loss, and postoperative scar hyperplasia. Particularly in pediatric patients, such procedures risk impairing mammary gland development. Our case adopted EAS technology, establishing an artificial intermuscular tunnel through a concealed 2-cm incision along the anterior axillary fold. The endoscopic magnification enabled complete exposure of tumor margins, achieving precise dissection. Studies demonstrate that EAS facilitates minintermuscular angiolipomaly invasive management of deep-seated lesions in complex anatomical regions, significantly reducing muscular trauma, intraoperative hemorrhage, and improving cosmetic outcomes (11, 12).

LTPRA utilizes 60–70°C radiofrequency energy to disintegrate cellular structures while achieving immediate hemostasis. Its low-temperature characteristics minimize thermal damage to adjacent muscles and nerves (13). In this procedure, the plasma scalpel enabled layer-by-layer ablation of tumor-adherent tissues with merely 15 ml intraoperative blood loss, and no postoperative edema or aggravated pain was observed. Compared to conventional electrocautery or ultrasonic scalpels, plasma technology proves particularly advantageous for vascular-rich AL resection by effectively controlling hemorrhage from large-caliber vessels at tumor pedicles.

For pediatric chest wall AL, treatment must balance radical tumor excision with functional preservation. Six-month follow-up revealed inconspicuous axillary scarring, maintained bilateral thoracic symmetry, and unaffected mammary development, confirming the safety and aesthetic superiority of endoscopic combined with LTPRA.

## 4 Conclusion

Endoscopic-assisted LTPRA provides a therapeutic strategy integrating minintermuscular angiolipomaly invasive features, procedural safety, and favorable cosmetic outcomes for intermuscular angiolipoma of the chest wall, particularly applicable to pediatric populations and cases with intricate anatomical configurations. Future prospective multicenter studies are warranted to validate its long-term therapeutic efficacy (especially regarding recurrence rate control) and generalizability to deep-seated soft tissue tumors.

## Data Availability

The original contributions presented in the study are included in the article/Supplementary Material, further inquiries can be directed to the corresponding author.
